# Consequences of the Trans-Atlantic Slave Trade on Medicinal Plant Selection: Plant Use for Cultural Bound Syndromes Affecting Children in Suriname and Western Africa

**DOI:** 10.1371/journal.pone.0112345

**Published:** 2014-11-05

**Authors:** Tessa Vossen, Alexandra Towns, Sofie Ruysschaert, Diana Quiroz, Tinde van Andel

**Affiliations:** 1 Leiden University, Leiden, The Netherlands; 2 Naturalis Biodiversity Center, Leiden University, Leiden, The Netherlands; 3 WWF Guianas, Paramaribo, Suriname; 4 Wageningen University, Biosystematics Group, Wageningen, The Netherlands; Cairo University, Egypt

## Abstract

Folk perceptions of health and illness include cultural bound syndromes (CBS), ailments generally confined to certain cultural groups or geographic regions and often treated with medicinal plants. Our aim was to compare definitions and plant use for CBS regarding child health in the context of the largest migration in recent human history: the trans-Atlantic slave trade. We compared definitions of four CBS (*walk early*, *evil eye*, *atita* and *fontanels*) and associated plant use among three Afro-Surinamese populations and their African ancestor groups in Ghana, Bénin and Gabon. We expected plant use to be similar on species level, and assumed the majority to be weedy or domesticated species, as these occur on both continents and were probably recognized by enslaved Africans. Data were obtained by identifying plants mentioned during interviews with local women from the six different populations. To analyse differences and similarities in plant use we used Detrended Component Analysis (DCA) and a Wald Chi-square test. Definitions of the four cultural bound syndromes were roughly the same on both continents. In total, 324 plant species were used. There was little overlap between Suriname and Africa: 15 species were used on two continents, of which seven species were used for the same CBS. Correspondence on family level was much higher. Surinamese populations used significantly more weedy species than Africans, but equal percentages of domesticated plants. Our data indicate that Afro-Surinamers have searched for similar plants to treat their CBS as they remembered from Africa. In some cases, they have found the same species, but they had to reinvent the largest part of their herbal pharmacopeia to treat their CBS using known plant families or trying out new species. Ideas on health and illness appear to be more resilient than the use of plants to treat them.

## Introduction

Perceptions of illness and health vary widely among different cultures. Syndromes that are perceived as an “illness” in certain cultures are not always diseases with well-defined medical equivalents. Local concepts of illness are an important feature in the health perception and medicinal practices of different cultural groups. These include cultural bound syndromes (CBS), defined as: “a group of folk illnesses, each of which is unique to a particular group of people, cultural, or geographical area” [1]. These ailments mostly consist of a variety of symptoms which causes are explained by cultural or magical beliefs [2]. CBS are known to persist in populations even after migration to an area where their beliefs about health and illness are not shared with the local community. The well-known Latin-American CBS *susto* (fright), defined as a person who is believed to have lost his/her soul and became ill as a result of fright by an unexpected accident, is also known among the Hispanic population in the United States [3], [4]. The practise of drinking bitter tonics, an African medicinal mixture used to enhance male sexual power and prevent disease by “making the blood bitter”, has survived two migratory moves: it is common among descendants of African slaves in the Americas and among Caribbean migrants in Europe [5].

The treatment of CBS often includes the use of medicinal plants [6]. Migrating to a novel geographical area means that the migrant population faces a change in floristic environment. Ethnobotanical research helps to interpret how people adapt to a new environment through the documentation of plant use patterns. In general, two strategies are used by migrants: (1) treating conditions with known plant species, which may be brought from the homeland or are also present in the new environment and (2) adapting plant use and resorting to novel species in the new environment that may be similar to known species from the homeland [7]. In this study, we compare Surinamese and African populations to study CBS and associated plant use in the context of the largest migrations in recent human history: the trans-Atlantic slave trade. Although the enslaved Africans could not bring many plants from their homeland, their traditional ideas about health and sickness and their plant knowledge were transferred with them across the ocean [5], [8].

**Figure 1:**
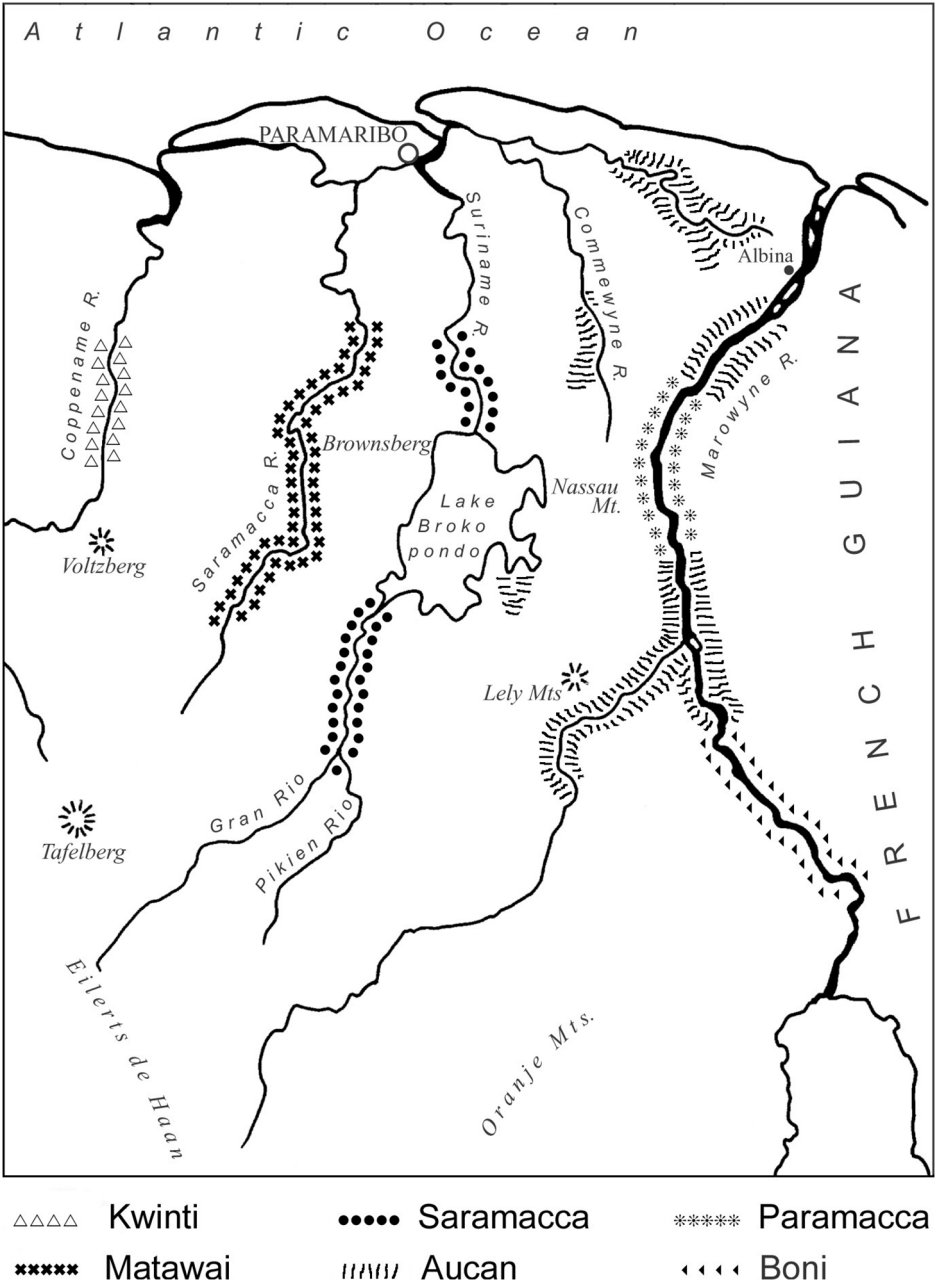
Maroon tribes of Suriname. Illustration by H. Rypkema. Source: Naturalis Biodiversity Center.

Between 1668 and 1823, about 300,000 African slaves were brought to the Dutch colony of Suriname [9], [10]. Most people came from the area between southern Gabon and northern Angola, followed by people from Ghana and Bénin [9]. Because of the brutal conditions on the coastal plantations, thousands of slaves escaped into the interior forests where they established independent communities, known as Maroons. These communities became so successful that by 1760 the colonial government was forced to sign peace treaties with them [10]. Today, six Maroon tribes with a total population of 72,553 exist in Suriname [11], of which the Aucan and Saramaccan populations are the largest. Most of them still live in semi-independent communities along the main rivers (Figure 1). After the abolition of slavery in 1863, former slaves who remained on the coastal plantations settled mostly in the capital Paramaribo. Their descendants are now generally known as Creoles. The use of medicinal and ritual plants was an important aspect in the daily life of the African captives in the Americas [8], [12]. However, tropical forests in Africa and the Americas differ substantially in their floristic composition. They share less than 1% of their total number of species, including domesticated exotics and pantropical weeds [13]. Thus, the enslaved Africans were forced to adapt their medicinal plant use to a completely new environment in order to survive [5], [14].

Africans can be considered relatively recent migrants in America, and the healing flora of their descendants often consists largely of pantropical weeds and domesticated exotics, and contains few native trees [15]. Recent research [5], however, shows that enslaved Africans in Suriname were very flexible in reinventing their herbal pharmacopeia. When faced with new diseases, both Maroons and Creoles learned from local Amerindians and Europeans which plants to use in their treatments but also practised trial and error to discover new herbal remedies [16]. For specific cultural bound ailments, however, they had limited opportunities to exchange ethnobotanical information with non-Africans, as these groups were less likely to share these health concepts. Therefore, it is expected that for the treatment of CBS, slaves in the New World relied on their own African knowledge and searched for Surinamese plants that were either botanically related or otherwise similar to the African species they used before their trans-Atlantic journey. After the Maroons fled from slavery in the 17th and 18th century, there was relatively little contact between plantation slaves and the different Maroon groups, so possibly plant use has diverged since the time these groups separated.

In this study, we focused on four cultural bound syndromes that were previously recorded among people of African descend in Suriname and in West and Central Africa. All four CBS concern health conditions of young children: *walk early*, *evil eye*, *atita* and *fontanels*. We investigated the definitions of these CBS across the Atlantic and their associated plant use by Afro-Surinamers (Creoles, Aucan and Saramaccan Maroons), and people from several ethnic groups in the countries where the Dutch bought their enslaved ancestors: Ghana, Bénin and Gabon. Our research focused on the following questions: 1) How are the four CBS (*walk early*, *evil eye*, *atita* and *fontanels*) defined among Afro-Surinamers and their ancestral African populations? and 2) How similar is the medicinal plant use regarding these CBS in Suriname, Ghana, Bénin and Gabon? We expected the definitions of the CBS and plant use to be similar across the Atlantic. As weedy and domesticated plants were probably the only plants enslaved Africans recognized from their motherland [15], we assumed that more of such species were used in Suriname than in Africa.

## Materials and Methods

### 2.1 Fieldwork

**Figure 2:**
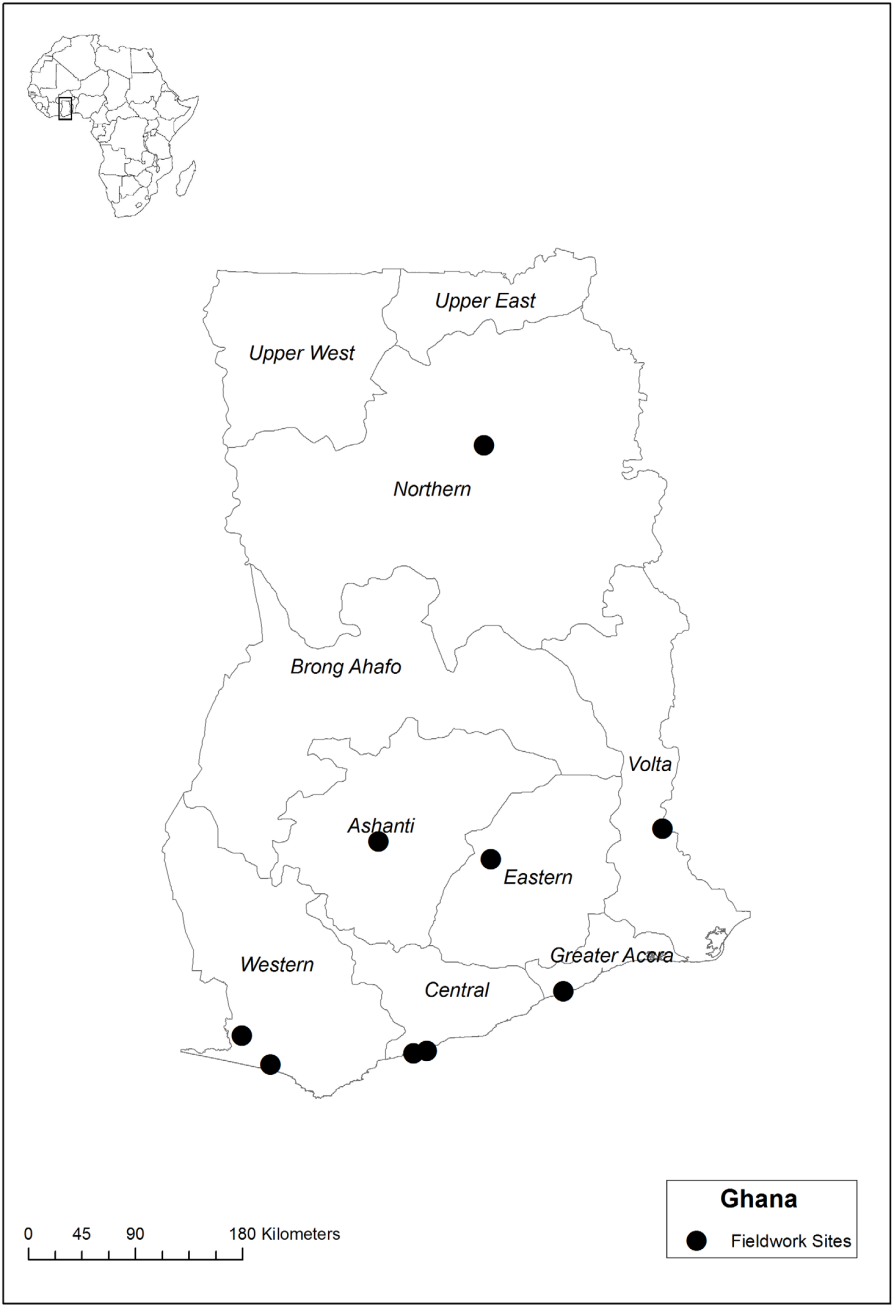
Fieldwork locations in Ghana.

**Figure 3:**
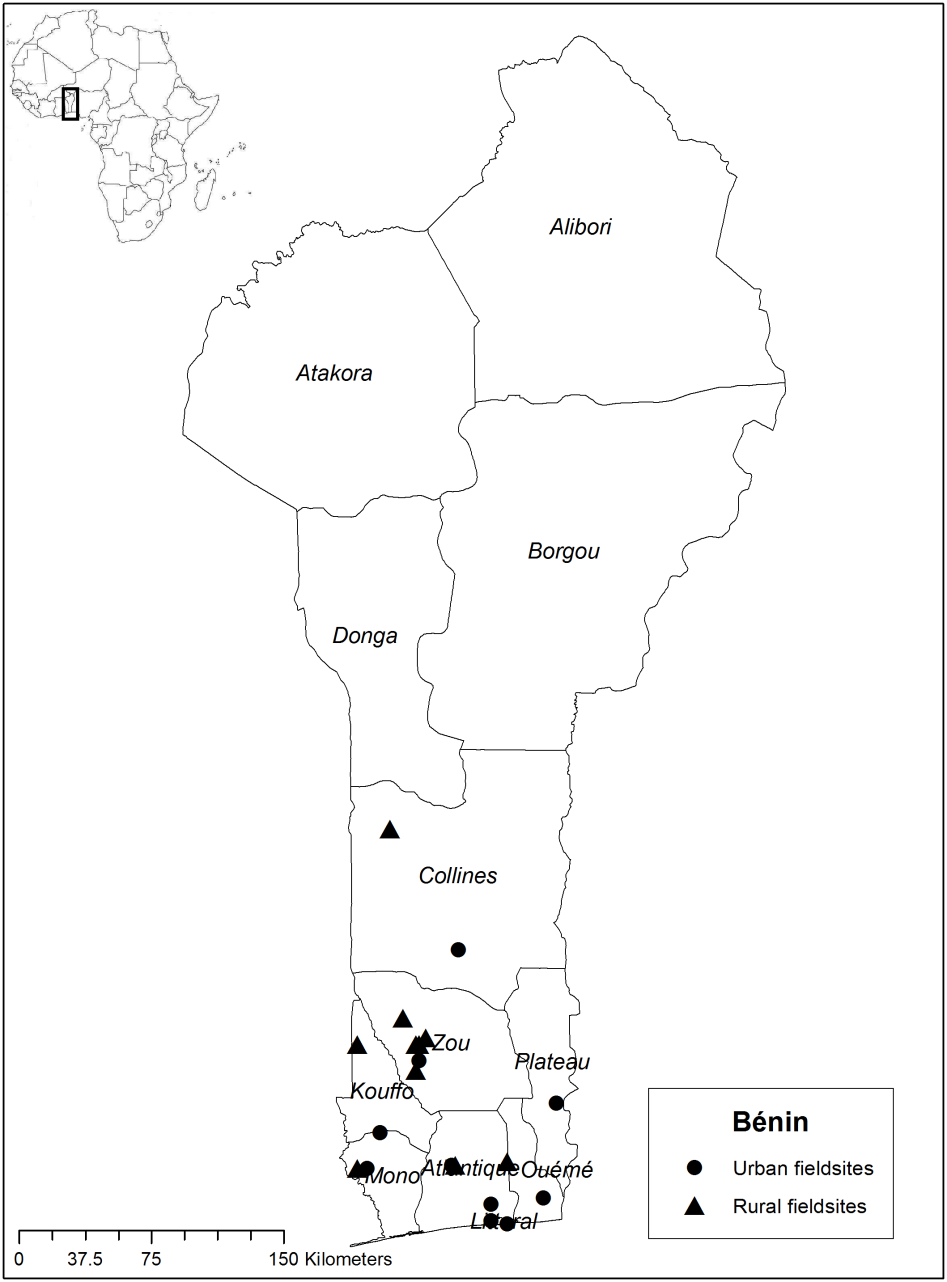
Fieldwork locations in Bénin. Source: Towns et al. (2014) [20].

**Figure 4:**
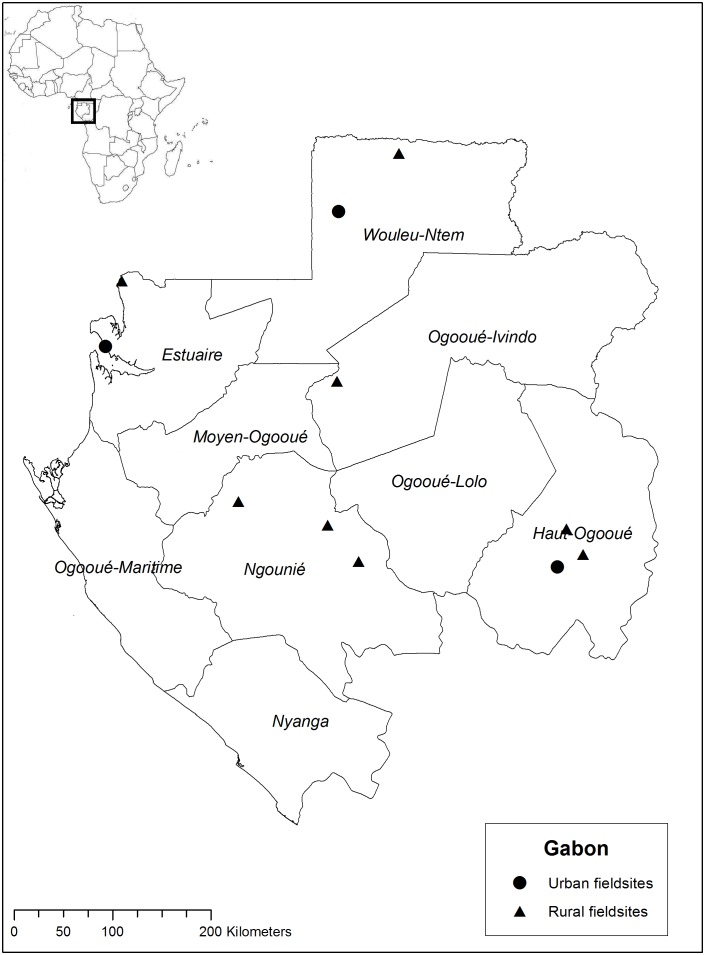
Fieldwork locations in Gabon. Source: Towns et al. (2014) [20].

Data on plant use for the four CBS were retrieved from ethnobotanical inventories on general medicinal plant use in Suriname among Saramaccan Maroons in Brownsweg in 2004–2006, among Creoles in Paramaribo and Aucans in Bigiston in 2006 [17]. Information on plant use in Ghana came from fieldwork in 2010 [18]. Data were further extracted from ethnobotanical interviews conducted on ritual plant use and child care in Bénin (2011) and Gabon (2012) [19], [20]. African fieldwork locations are indicated in Figure 2, 3 and 4. Additional data were obtained from literature on Surinamese Creoles [21]–[23] and Ghanaians [24]–[27]. These data were complemented with fieldwork and interviews specifically focused on CBS in childcare among 25 Aucan women conducted in June and July (2013) in the village of Mooitaki, Tapanahoni river, southeast Suriname and with Saramaccan herbal medicine vendors in Paramaribo [28]. In all four countries, we started our research at the herbal markets, taking time to familiarize ourselves with commonly utilized species, local illnesses and healthcare practices. From these initial market contacts, we utilized snowball sampling to identify women from surrounding urban and rural communities. We also contacted family members of African and Surinamese migrants in the Netherlands, who invited us to their village. Within the rural communities, we first discussed the nature of our research with village elders and school teachers, after which participants were selected and/or volunteered. Our main form of data collection was an interview on ethnobotanical practices related to childcare, with questions on species and recipes for specific illnesses and definitions of folk illnesses [20]. Subsequent to the interviews, plants mentioned by our informants were processed into botanical vouchers using standard collection methods. During market surveys, plants of interest were purchased directly from the vendors. Duplicates were deposited at the National Herbarium of Suriname (BBS), the Ghana Herbarium (GC), the Herbier National du Bénin (BEN), the Herbier National du Gabon (LBV) and Naturalis Biodiversity Center (L). Scientific and author names were checked using The Plantlist [29]. Additionally, three interviews were held with medical staff in Paramaribo and Mooitaki to assess their knowledge of symptoms and causes of CBS among Afro-Surinamers. The medical specialists were first contacted by email and after their written agreement, we made an appointment in their hospital to conduct the interviews. The information provided by medical staff was anonymized.

### 2.2 Data analysis

We constructed a database with lists of plants used per cultural bound syndrome in the three African countries (Ghana, Bénin and Gabon) and Suriname, divided into Creoles, Saramaccans and Aucans (Table S1). We refer to these six categories as “populations” throughout the paper, although we are aware that the data from the African countries came from several different ethnic groups. All plant species and families were entered in a presence–absence data matrix with species in rows and CBS per population in columns. All plants used for a certain CBS per population were used as the sample unit in our analysis. To assess the similarity in plant use between countries and populations, we performed a Detrended Component Analysis (DCA) on species and family level. Unidentified plants were included as separate species in the analysis on species level, but excluded on family level. Specimens only identified to family were included in the analysis on both levels. We plotted the results of our DCA analysis on the two main axes that caused the distribution of the data to visualise potential overlap and variation in plant use by the six populations. All analyses were performed in PC-ORD 5.0. To compare the proportion of wild and domesticated species used by the six populations, we constructed another matrix in which we listed for each plant whether it was weedy, domesticated or wild (which included plants taken from the forest to the village to be cultivated) and whether it occurred in Suriname and/or the three African countries (Table S2). This information was observed in the field and retrieved from literature [17], [30]–[35]. To estimate whether the Surinamers used a higher proportion of domesticated and weedy species than the Africans, we performed a Wald Chi-squared test using IBM SPSS 19.0. The map for Ghana was created in ArcGIS 10.1 using open source geospatial data from DIVA-GIS. The Benin and Gabon maps were reproduced from Towns et al [20].

### 2.3 Ethics statement

The authors adhered to all components of the Code of Ethics of the International Society of Ethnobiology [36] to ensure ethical conduct in the design, implementation, and publication of their research. More specifically, we shared full disclosure on the nature of the research, received prior informed consent from all participants, provided monetary compensation appropriate to the local norms for participants’ involvement in the research, and ensured participants’ confidentiality by anonymizing their identities in databases and publications. For all interviews conducted in the field, prior oral consent was obtained. When informants were literate, they signed a written consent form. In Bénin, the authors obtained a formal invitation from the Faculté des Sciences Agronomiques and a research permit from the Faculté des Sciences et Techniques, both at Universite d’Abomey-Calavi. In Gabon, they received a letter of invitation and research permit from CENAREST and authorization from the Agence Nationale des Parcs Nationaux (ANPN) to enter the National Parks. They also acquired the necessary export permits from IPHAMETRA in Gabon from the Service de la Protection des Vegetaux et du Control Phytosanitaire, Ministre de l’Agriculture, de l’Elevage et de la Peche in Benin for all botanical material collected. In Ghana and Suriname, they received approval for plant collection and export of herbarium samples from the Suriname Forest Service, and the Ministry of Food and Agriculture (Plant Protection and Regulatory Services Directorate) of the Republic of Ghana. In order to receive research permits in each of the countries where we conducted fieldwork, we had to submit a full research proposal which included a detailed consent protocol. Digital copies of each of these permits are available upon request. Naturalis Biodiversity Center does not have an ethics committee or an institutional review board, but has formal partnerships with all the institutes mentioned above. Our partners ensured us that no additional ethical approval or permits were required.

## Results

### 3.1 Trans-Atlantic definitions of cultural bound syndromes and treatments

#### 3.1.1 Walk early

According to our Surinamese Maroon, Béninese and Gabonese informants, a baby should walk early to prove that the child is active, curious and strong, and its mother will have more time for her own activities. This CBS is often treated by using plants to make the baby walk sooner. We did not find evidence of Surinamese Creoles using stimulants to make their children walk early. Aucan mothers mentioned that the herbs they used not only served to make the baby walk early, but also made it fat and strong. These herbs were called “*táanga sikin uwii*”: herbs to make the body (litt. skin) strong. All African populations rubbed plants on legs and joints to make children walk sooner, whilst Maroons used many herbal baths and tended to softly hit the baby’s legs with brooms, plants or other items. Drinking plant decoctions was mentioned on both continents. The use of locally named “vaccinations” -rubbing juice from herbs into skin incisions - was only recorded for Gabon. The use of rectal insertions (“bentua”) to enhance walking was only recorded for Ghana.

#### 3.1.2 Evil eye

Evil eye in Suriname is called “*ogri ai*” (litt. bad eye) or “*sama mofu*” (litt. someone’s mouth; meaning someone’s curse). All Surinamese groups described evil eye as a sickness that babies, and sometimes adults as well, could get when a person looked too strongly at them or admired them too much while secretly being jealous. This could affect the victim’s wellbeing, whether intended or not. To cure or prevent evil eye, most Surinamers bathed the baby with water containing Reckitt’s Blue. People also used herbal baths, jewellery (beads and bracelets) and rubbed *asafoetida* (a foul smelling substance made of the roots of the Indian plant *Ferula asafoetida*) in the baby’s hair. Reckitt’s Blue was sometimes also rubbed on the head, behind the ears, in palms and footpads, and between the buttocks of the child. In the three African countries, the specific term evil eye was not mentioned. However, in Ghana, herbal medicine or baths were applied to protect children from evil or danger, while in Gabon, baths and infusions were used in protection against “*djedimikoki*” or “*fussile nocturne*” (nocturnal rifle), a similar concept of ill-health or misfortune caused by jealousy.

#### 3.1.3 Atita

The CBS atita was known by all six populations, although under different names. The term “*atita*” was used on both sides of the Atlantic, by Saramaccan and Aucan Maroons and the Béninese population. Surinamese Creoles addressed this CBS as “*zuurte*” or “*suri*” (litt. sourness), the Gabonese call it “*fesse-rouge*” (red buttocks), and the Ghanese population used the terms “*tumo koko*”,”*djudjuma*” or “*djindjuma*”. In Suriname, the children’s ailment “*atita*” was mainly defined by the appearance of the baby’s faeces, which was said to be yellow, smell sour, and come like diarrhoea with small seed-like balls. Almost all Surinamese and Africans mentioned the simultaneous occurrence of diaper or body rash (especially in the face, armpits and groin). African mothers did not mention the colour, texture and scent of the baby’s faeces. Treatments included herbal baths, plant decoctions, and applying plant mixtures (only Africans) or oil (only Surinamese) on the rash. Pharmacies in Paramaribo also sold medicinal oils to treat *atita*. Possible causes of *atita* included the mother eating too much sweet foods, like peanuts (Bénin), bananas or sweet potatoes (Aucans). Some Gabonese and Aucan mothers said the illness was caused by God. A few Aucan women mentioned that all children were born with *atita* and remedies should be given preventively directly after birth. On Creole internet fora [23], food allergy was sometimes reported as a cause for *atita*, but also liver or digestion problems and intestinal worms (also mentioned as a cause in Bénin). Gabonese informants said that “*fesse rouge*” was caused by the naked baby sitting on dirt floors that were polluted by microbes. Some Aucans suggested that pregnant women could get *atita* too. If the expecting mother had the condition while pregnant, the newborn would surely get *atita* as well.

#### 3.1.4 Fontanels

The fontanel, a soft membranous gap between the cranial bones of an infant skull, was closely monitored by mothers in both Africa and Suriname. The failure of the fontanels to close was generally thought to cause several illnesses. Aucans called the fontanel “*bwébwé*” (possibly an African term, although we were not able to identify its African origin) and applied coconut oil on it when the baby had a cold, just like Creole and Gabonese mothers. Aucan women said the fontanel had to move up and down, so they knew the child’s heart was beating. To stimulate these movements, they chewed maize or *Aframomum melegueta* seeds and spat these on the fontanel. In Gabon, kola nuts (*Cola* sp.) were chewed and spat on the baby’s head, while ground *A. melegueta* seeds were rubbed on the child’s palate. Some Aucan mothers also mentioned “*amon*”, a black substance on the baby’s head or fontanel, which they carefully scraped off with coconut oil. In Ghana, a beating fontanel was treated by applying herbal paste on it. Béninese mothers applied pastes on the fontanel in order to secure its correct closure. Other African treatments included herbal baths, oral decoctions and washing the head. Surinamers did not make a cultural connection between the fontanel and the baby’s palate. No records on herbal medicine for the fontanel were found among Saramaccans.

When asked, Aucan mothers stated they would not seek medical attention if their child was suffering from a CBS, partly also because they were convinced that medical staff did not know about these cultural ailments or that they had no cure for them. However, if symptoms appeared to be severe (e.g. high fever), they took their child to a doctor to treat the symptoms.

### 3.2 Plant use

**Figure 5:**
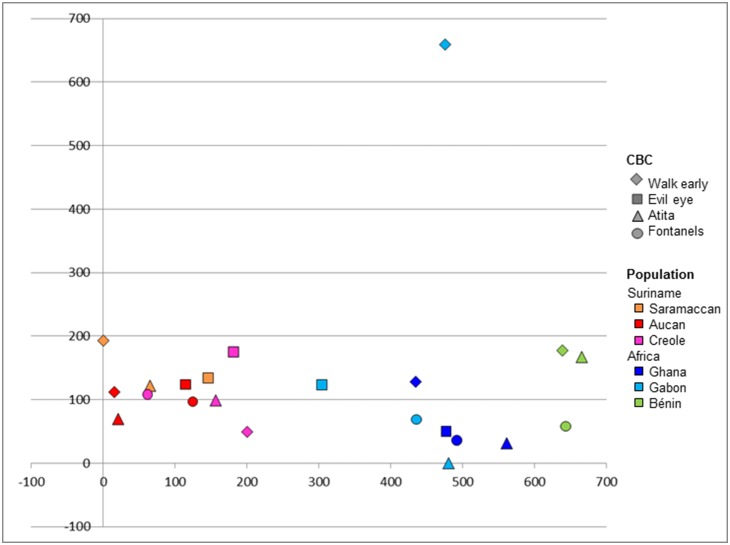
Scatter plot showing similarity in plant use on species level (n = 324). Datapoints indicate plants used for a specific CBS by a specific populations. Clustered datapoints indicate similarity in plant species used. Axes do not represent variables but serve to visualize variation and similarity in plant use.

**Figure 6:**
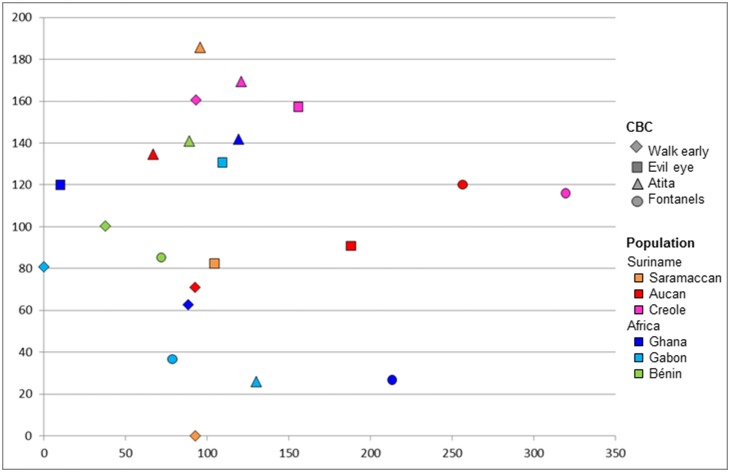
Scatter plot showing similarity in plant use on family level (n = 81). Datapoints indicate plant families used for a specific CBS by a specific populations. Clustered datapoints indicate similarity in plant families used. Axes do not represent variables but serve to visualize variation and similarity in plant use.

In total, 324 plant species were used by the six populations for the four CBS, of which 146 were recorded in Suriname and 193 in Africa. All plant species are listed with their scientific names, family and uses in Table S1. Fifteen species (4.6%) were used on both continents, of which seven species were employed for the same CBS: *Paullinia pinnata* (walk early, evil eye), *Aframomum melegueta* (fontanels), *Scoparia dulcis* and *Cecropia peltata* (both for walk early), *Eclipta prostata*, *Musa* sp. and *Senna alata* (all for *atita*). There was little overlap in plant species used between the six populations in Africa and Suriname (Figure 5). Categories for which no plant species were used (Table 1) were left out of the analysis. There was substantial variation within the African populations (as can be seen from the outlier represented by plants used for walk early in Gabon), while plant use among Surinamese Maroons and Creoles was much more similar on species level. Plant use on species level clustered according to geographical location rather than per cultural bound syndrome. This implies that each population, on both sides of the Atlantic, adapted their plant use to what plant species were available in their direct surroundings. If we consider plant family use per population and CBS (Figure 6), there is much more overlap and almost no division between Africa and Suriname. Families that were recorded most frequently for CBS (Table 2) are common medicinal plant families (e.g. Fabaceae, Euphorbiaceae, Asteraceae) that occur on both sides of the ocean. This suggests that when Africans arrived in the New World, they continued to use the same plant families as they did in the Old World.

**Table 1:** The number of plant species per cultural bound syndrome per population.

	Saramaccans	Aucans	Creoles	Ghana	Bénin	Gabon
***Walk early***	60	37	1	20	38	29
***Evil eye***	35	20	15	11	0	1
***Atita***	33	32	11	2	39	30
***Fontanels***	0	7	2	4	35	28
**Total nr. of plant species** *	104	70	26	35	98	76
	Suriname			Africa		
	146			193		

*Summed single values surpass the total number of plant species, because species are often used for more than one CBS and may also overlap between populations. This is especially the case for Suriname.

**Table 2:** The most commonly used plant families, mentioned at least for one of the four CBS on each continent.

Family	Citation frequency*	
	Suriname	Africa
Fabaceae	8	8
Euphorbiaceae	8	6
Asteraceae	7	7
Arecaceae	6	7
Malvaceae	5	8
Sapindaceae	7	3
Rubiaceae	4	6
Piperaceae	8	2
Poaceae	8	1
Lamiaceae	2	7
Verbenaceae	6	2
Plantaginaceae	5	2
Myrtaceae	6	1
Annonaceae	3	4
Zingiberaceae	3	4

*Nr. of times mentioned in absence-presence data matrix, regardless of ethnic group or specific CBS.

### 3.3 Use of weeds and domesticated plants

Of all plants used in Suriname (n = 146), 54% were either weedy or domesticated species (Table 3). In African countries, this proportion was 32% (n = 193). People in Suriname used a significantly higher proportion of weedy species for CBS than Africans, but differences between Africa and Suriname with regard to the proportion of domesticated and wild plants were not significant (Table 3). This can partly be explained by the higher number of unidentified species recorded in Africa. Of the 43 plant species used for CBS in Africa that also occur in Suriname, only 15 were used for CBS as well; 87% of them were weedy or domesticated plants.

**Table 3:** Percentage of weedy, domesticated and wild species in Africa and Suriname.

Used in	Total	Weeds	Domesticated	Wild	Unidentified
**Africa**	193	41 (21%)	21 (11%)	132 (68%)*	38 (20%)
**Suriname**	146	56 (38%)	24 (16%)	123 (84%)	0 (0%)
**p-value**	-	0.001	0.138	0.068	
**Wald Chi-square**	-	11.656	2.203	3.337	
**Significance**	-	s	ns	ns	

*Percentages of wild, domesticated and unidentified (including species identified up to genus level for which domestication status is unknown). count up to 100%. Weeds are always wild species.

## Discussion

### 4.1 Trans-Atlantic definitions of cultural bound syndromes

Although fontanels were closely monitored and treated with herbal medicine by both African and Surinamese populations, the Surinamese did not address the shape of the fontanel (e.g. sunken or bulging) while defining the CBS. The African populations gave less clear descriptions of evil eye than the Surinamers, although jealousy-related child diseases were reported in Ghana and Gabon. The definitions of the CBS walk early and atita were strikingly similar across the Atlantic. The occurrence of *atita* and the importance of a child walking early was documented for all six populations addressed in this study. Even the Béninese Fon term *atita* was retained among Aucans. In many African cultures, such as the Kwahu in Ghana [37] or the Yoruba in West Africa [38], partners should only resume their sex life when their youngest child reaches a certain developmental state, such as being able to walk or teething [37]–[43]. Although such traditions help birth spacing between children [38], a mother often uses medicinal plants to encourage her child to walk, so she can resume her sex life and prevent her husband from starting an affair with another woman [40], [41]. None of our Surinamese and African informants, however, related the parents’ sex life to the importance of a child to walk early.

Evil eye is a well-known cultural condition not only limited to Afro-American or African cultures [44]–[47], but also mentioned in Hebrew religious texts [48], the Quran [49] and the Bible [44], [50]. Described to be caused by jealousy, or a “strong look” [45], the evil eye is believed to particularly affect young children [51]. Lists of symptoms include weakness, diarrhoea, vomiting, crying, reduced appetite and fever [21], [22], [51]. In Ghana, a condition similar to evil eye was described as “*asram*” [52], defined as a sickness affecting young babies, caused by intended or unintended jealousy. Treatments included herbal medicine or baths. Our informants did not mention the term *asram*. Although Béninese babies could suffer from a certain bewitchment from parents and relations [53], no literature on jealousy-related conditions were reported for this country.

Only two records of *atita* as a CBS were found in literature [20], [54]. In biomedical literature, sour-smelling defecation, a symptom of *atita*, has been reported as a consequence of malabsorption of saccharides due to lactase deficiency [55], [56]. According to a doctor and a paediatrician at the Academic Hospital of Paramaribo we interviewed, *atita* was not medically defined. They doubted whether the condition was caused by lactose intolerance, because most babies who suffered from *atita* were very young and breastfed. They suggested that *atita* was linked to the baby’s intestinal flora which had to adapt to the uptake of proteins from breast milk. The grains in the faeces could be undigested protein matter, caused by a not fully developed bacterial culture in the bowels.

The belief of some of our Surinamese and African informants that spirits could enter through the fontanel, or that the fontanel was some sort of ‘head road’ was reported earlier [40], [57]. In Ghana, problems with the fontanel were said to be linked to cerebral diseases [24]. A condition named “*puni*”, characterized by changes in the baby’s skull, possibly relating to the fontanels, was also reported in this country [52]. Many records on CBS mention abnormalities of the fontanel amongst the main symptoms, for example a “fallen fontanel”, a condition resulting in feeding difficulties, diarrhoea, fever and weakness. This condition is believed to be caused by a fall or by pulling the nipple out of the baby’s mouth too suddenly, causing the baby’s palate and the fontanel to “fall” [51], [58]–[60]. In this study, African mothers paid attention to the baby’s palate in respect to the fontanel, whereas Surinamese mothers did not. Fontanel abnormalities can be an indication of more serious conditions: a sunken fontanel can indicate dehydration [60], [61], while a bulging fontanel can indicate meningitis [62], [63] or vitamin A deficiency [64].

### 4.2 Plant use

As the Surinamese groups used significantly more weedy species than the African ones but roughly the same proportion of domesticated plants, our data partially confirm our hypothesis that enslaved Africans in the New World continued to use “familiar” species (weeds and domesticates) in favour over finding new cures [15], [64]. The large percentage of such weeds and domesticates used by African mothers rather suggests that women in general search for herbal medicine in the limited space around their houses and villages, areas often abundant in domesticated and weedy species [66], [67].

The little overlap on species level between African and Surinamese plant use for CBS suggest that enslaved Africans did not limit themselves to previously known plants. Not all 43 ‘African’ CBS plants that also occur in Suriname were used similarly. Previous research on the use of bitter tonics across the Atlantic showed that Africans in the New World have used their surrounding new flora in a creative way. Apart from looking for similar African families, weeds and domesticates, they also learned to use new, Neotropical plants for their African health issues [5]. Similar to the case of the bitter tonics, plant use for children’s CBS showed much more variation in Africa than in Suriname. The position of the outlier in Gabonese plant use suggests an even wider diversity of plant use, but data from most West and Central African countries on the four CBS are lacking.

Rather than migration or geographical separation, time is also a factor in the dynamic character of medicinal plant use. In the 1760s, the Swedish plantation owner and botanist Gustav Dahlberg collected a Surinamese plant named “*atita*”, which he described as being used medicinally to cure worm infections in small children [68], [69]. Lanjouw and Uittien [70] identified Dahlberg’s plant as *Cleome gynandra*, but also mentioned another Surinamese herb with the name “*atita*”: *Oldenlandia herbacea*. In 2013, only one Aucan mother recognized *O. herbacea* as an “*atita* herb”, although it was growing abundantly in her village. The genus *Cleome* is no longer used medicinally in the country. Nowadays, *Nepsera aquatica* is sold as the main “*atita* herb” on the medicinal plant market in Paramaribo by Saramaccan Maroons [17], while Aucans referred to *Senna chrysocarpa* as the most important “*atita* herb”. In Bénin, we encountered a plant called “*atita ma*” (*atita* leaf, *Cremaspora triflora*), employed in the treatment of another illness than *atita*, but probably used for this ailment in the past. Apparently, favoured species for CBS in a certain period of time may be forgotten later on. This could explain why, of the 43 plants in our database that occur both in Africa and Suriname, only 15 were used for CBS on both continents. Despite the limited similarity in plant use between the two continents, seven of the 15 plant species used on both continents were employed for the same CBS, indicating that certain plant use has survived the dynamic changes over time and space, implying their importance in both the African and Surinamese culture.

## Supporting Information

Table S1 **Species in the plant database: family, scientific name, CBS for which used, and population.** CBS are *walk early* (W), *evil eye* (E), *atita* (A) and *fontanels* (F). Populations are Saramaccan (SA), Aucan (AU), Creole (CR), Ghana (GH), Bénin (BE) and Gabon (GA).
(DOCX)

Table S2 **Matrix with plant species used per CBS and population, with indications of wild, weedy and domestic status, as well as occurrence per species.**
(XLS)
