# Supplementary material for: Consequences of the Trans-Atlantic Slave Trade on Medicinal Plant Selection: Plant Use for Cultural Bound Syndromes Affecting Children in Suriname and Western Africa
Source: PLoS One. 2014 Nov 5;9(11):e112345. doi: 10.1371/journal.pone.0112345 (PMC4221471; doi:10.1371/journal.pone.0112345)
Supplement: Table S1 — Species in the plant database: family, scientific name, CBS for which used, and population. CBS are walk early (W), evil eye (E), atita (A) and fontanels (F). Populations are Saramaccan (SA), Aucan (AU), Creole (CR), Ghana (GH), Bénin (BE) and Gabon (GA). (DOCX) [file pone.0112345.s001.docx]

| **Family** | **Scientific name** | **CBS** | **Population** |
| --- | --- | --- | --- |
| Acanthaceae | *Acanthus montanus* (Nees) T.Anderson | W | GA |
| Acanthaceae | *Justicia calycina* (Nees) V.A.W. Graham | W | SA |
| Acanthaceae | *Justicia flava* (Forssk.) Vahl | F | BE |
| Acanthaceae | *Justicia pectoralis* Jacq. | W | SA |
| Acanthaceae | *Justicia secunda* Vahl | W | SA |
| Amaranthaceae | *Alternanthera brasiliana* (L.) Kuntze | W | SA |
| Amaranthaceae | *Alternanthera pungens* Kunth | W | BE |
| Amaranthaceae | *Amaranthus blitum* L. | E | SA |
| Amaranthaceae | *Cyathula prostrata* (L.) Blume | W  F | SA  GA |
| Amaranthaceae | *Dysphania ambrosioides* (L.) Mosyakin & Clemants | E  F | GH  BE |
| Amaranthaceae | *Pfaffia glomerata* (Spreng.) Pedersen | W | SA |
| Amaranthaceae | *Pupalia lappacea* (L.) Juss. | F | BE |
| Amaryllidaceae | *Allium sativum* L. | E  A, F | CR  BE |
| Amaryllidaceae | Amaryllidaceae sp. | W | GA |
| Anacardiaceae | *Anacardium occidentale* L. | A | SA, AU |
| Anacardiaceae | *Lannea acida* A.Rich. | W | BE |
| Anacardiaceae | *Mangifera indica* L. | W  A | BE  GA |
| Annonaceae | *Annickia affinis* (Exell) Versteegh & Sosef | A | GA |
| Annonaceae | *Annona muricata* L. | W, E  E | SA  AU |
| Annonaceae | *Monodora tenuifolia* Benth. | W | BE |
| Annonaceae | *Uvaria chamae* P.Beauv. | A, F | BE |
| Annonaceae | *Xylopia aethiopica* (Dunal) A.Rich. | W | BE |
| Apiaceae | *Eryngium foetidum* L. | E | SA, AU, CR |
| Apiaceae | *Ferula asafoetida* H. Karst. | E, F  F | AU  CR |
| Apiaceae | *Foeniculum vulgare* Mill. | A | CR |
| Apiaceae | *Pimpinella anisum* L. | A | CR |
| Apocynaceae | *Allamanda cathartica* L. | E | CR |
| Apocynaceae | *Carissa spinarum* L. | W | BE |
| Apocynaceae | *Marsdenia latifolia* (Benth.) K.Schum. | W | GH |
| Apocynaceae | *Pergularia daemia* (Forssk.) Chiov. | F | BE |
| Apocynaceae | *Rauvolfia vomitoria* Afzel. | F | BE |
| Apocynaceae | *Secamone afzelii* (Roem. & Schult.) K.Schum. | A, F | BE |
| Araceae | *Philodendron hederaceum* (Jacq.) Schott | W  E | AU  SA |
| Arecaceae | Arecaceae sp. | W | BE |
| Arecaceae | *Cocos nucifera* L. | E, A  A, F  F | SA  AU  CR |
| Arecaceae | *Elaeis guineensis* Jacq. | W  A  F | GH  BE, GA  BE, GA |
| Arecaceae | *Mauritia flexuosa* L.f. | W | SA |
| Arecaceae | *Raphia hookeri* G.Mann & H.Wendl. | F | GH, BE |
| Asparagaceae | *Asparagus warneckei* (Engl.) Hutch. | F | GA |
| Asparagaceae | *Dracaena fragrans* (L.) Ker Gawl. | W | GA |
| Asteraceae | *Acanthospermum hispidum* DC. | W | BE |
| Asteraceae | *Acmella caulirhiza* Delile | F | GA |
| Asteraceae | *Bidens pilosa* L. | W | GA |
| Asteraceae | *Chromolaena odorata* (L.) R.M. King & H. Rob. | W, A  W, E, A | SA  AU |
| Asteraceae | *Cyanthillium cinereum* (L.) H.Rob. | W, A | BE |
| Asteraceae | *Eclipta prostrata* (L.) L. | E, A  A | SA  AU, GA |
| Asteraceae | *Elephantopus mollis* Kunth | A | SA |
| Asteraceae | *Emilia coccinea* (Sims) G.Don | W, A | GA |
| Asteraceae | *Launaea taraxacifolia* (Willd.) Amin ex C.Jeffrey | W | GH |
| Asteraceae | *Mikania micrantha* Kunth | A | SA |
| Asteraceae | *Mikania psilostachya* DC. | W | SA |
| Asteraceae | *Rolandra fruticosa* (L.) Kuntze | W  W, A | SA  AU |
| Asteraceae | *Sphaeranthus senegalensis* DC. | W | GH |
| Asteraceae | *Struchium sparganophorum* (L.) Kuntze | E | SA, AU, CR |
| Asteraceae | *Tilesia baccata* (L.) Pruski | W, E | SA |
| Asteraceae | *Tithonia diversifolia* (Hemsl.) A.Gray | A | GA |
| Asteraceae | *Unxia camphorata* L. f. | A  E | SA  GH |
| Balanophoraceae | *Thonningia sanguinea* Vahl | W | GH |
| Begoniaceae | *Begonia glabra* Aubl. | E | SA, AU |
| Bignoniaceae | *Crescentia cujete* L. | E | SA |
| Bignoniaceae | *Dolichandra unguis-cati* (L.) L.G.Lohmann | W | SA |
| Bignoniaceae | *Mansoa alliacea* (Lam.) A.H.Gentry | E | SA |
| Bignoniaceae | *Stereospermum kunthianum* Cham. | W | BE |
| Bignoniaceae | *Handroanthus serratifolius* (Vahl) S.O.Grose | W | AU |
| Boraginaceae | *Cordia curassavica* (Jacq.) Roem. & Schult. | E | SA, CR |
| Boraginaceae | *Cordia schomburgkii* A.DC. | E, A | SA |
| Boraginaceae | *Heliotropium indicum* L. | A | BE |
| Burseraceae | *Boswellia papyrifera* (Caill. ex Delile) Hochst. | A | BE |
| Cannabaceae | *Trema micrantha* (L.) Blume | W, A | AU |
| Cannabaceae | *Trema orientalis* (L.) Blume | W | BE |
| Chrysobalanaceae | Chrysobalanaceae sp. | W | GH |
| Chrysobalanaceae | *Couepia* sp. | A | SA |
| Chrysobalanaceae | *Licania* sp. | W | SA |
| Cleomaceae | *Cleome viscosa* L. | W | BE |
| Clusiaceae | *Garcinia* sp. CF | F | BE |
| Combretaceae | *Combretum aphanopetalum* Engl. & Diels | A | GA |
| Combretaceae | *Combretum collinum* Fresen. | A | BE |
| Combretaceae | *Combretum micranthum* G.Don | F | BE |
| Combretaceae | *Pteleopsis suberosa* Engl. & Diels | W, A | BE |
| Combretaceae | *Terminalia catappa* L. | A, F | GA |
| Combretaceae | *Terminalia glaucescens* Planch. ex Benth. | A | BE |
| Commelinaceae | *Commelina diffusa* Burm.f. | E  W | SA  AU |
| Commelinaceae | *Commelina erecta* L. | W, E | SA |
| Commelinaceae | *Palisota ambigua* (P.Beauv.) C.B.Clarke | W | GA |
| Commelinaceae | *Tripogandra serrulata* (Vahl) Handlos | W | AU |
| Connaraceae | *Cnestis ferruginea* Vahl ex DC. | W | GH |
| Convolvulaceae | *Evolvulus alsinoides* (L.) L. | E | GH |
| Convolvulaceae | *Ipomoea mauritiana* Jacq. | F | GA |
| Convolvulaceae | *Ipomoea pileata* Roxb. | W | GA |
| Convolvulaceae | *Ipomoea tiliacea* (Willd.) Choisy | A | AU |
| Convolvulaceae | *Merremia tridentata* (L.) Hallier f. | A, F | BE |
| Costaceae | *Costus scaber* Ruiz & Pav. | A | AU |
| Costaceae | *Costus* spp. | A, F | GA |
| Crassulaceae | *Bryophyllum pinnatum* (Lam.) Oken | W | BE |
| Cucurbitaceae | *Cucumeropsis mannii* Naudin | W | GA |
| Cucurbitaceae | Cucurbitaceae sp. | A | BE |
| Cucurbitaceae | *Kedrostis foetidissima* (Jacq.) Cogn. | A | BE |
| Cucurbitaceae | *Melothria pendula* L. | A | CR |
| Cucurbitaceae | *Momordica charantia* L. | E  A | GH  BE |
| Cyperaceae | *Cyperus prolixus* Kunth | W, E | AU |
| Cyperaceae | *Scleria secans* (L.) Urb. | W | SA |
| Ebenaceae | *Diospyros guianensis* (Aubl.) Gürke | E | SA |
| Euphorbiaceae | *Alchornea cordifolia* (Schumach. & Thonn.) Müll.Arg. | A | BE, GA |
| Euphorbiaceae | *Croton gratissimus* Burch. | E, A | GH |
| Euphorbiaceae | *Croton* sp. | F | GA |
| Euphorbiaceae | *Croton trinitatis* Millsp. | W, A | SA |
| Euphorbiaceae | *Euphorbia hirta* L. | W, A  A | SA  AU |
| Euphorbiaceae | *Euphorbia thymifolia* L. | E  A | SA, AU  SA, AU, CR |
| Euphorbiaceae | *Macaranga barteri* Müll.Arg. | W | GA |
| Euphorbiaceae | *Jatropha multifida* L. | A | BE |
| Euphorbiaceae | *Mabea piriri* Aubl. | E | SA |
| Euphorbiaceae | *Macaranga spinosa* Müll.Arg. | W | GA |
| Euphorbiaceae | *Mallotus oppositifolius* (Geiseler) Müll.Arg. | A | BE |
| Euphorbiaceae | *Maprounea africana* Müll.Arg. | W | GA |
| Euphorbiaceae | *Maprounea guianensis* Aubl. | W | SA |
| Euphorbiaceae | *Maprounea membranacea* Pax & K.Hoffm. | W | GA |
| Euphorbiaceae | *Plagiostyles africana* (Müll.Arg.) Prain | W | GA |
| Euphorbiaceae | *Ricinus communis* L. | F | AU |
| Fabaceae | *Acacia erythrocalyx* Brenan | W | BE |
| Fabaceae | *Acacia sieberiana* DC. | W | BE |
| Fabaceae | *Acacia tenuifolia* (L.) Willd. | W | SA |
| Fabaceae | *Albizia ferruginea* (Guill. & Perr.) Benth. | F | BE |
| Fabaceae | *Andira* sp. | W | AU |
| Fabaceae | *Arachis hypogaea* L. | W  F | SA, AU  GA |
| Fabaceae | *Bauhinia guianensis* Aubl. | W | SA |
| Fabaceae | *Bocoa prouacensis* Aubl. | W | SA |
| Fabaceae | *Caesalpinia bonduc* (L.) Roxb. | A | BE |
| Fabaceae | *Caesalpinia pulcherrima* (L.) Sw. | A | BE |
| Fabaceae | *Crotalaria micans* Link. | E | SA |
| Fabaceae | *Crotalaria retusa* L. | F | BE |
| Fabaceae | *Cynometra megalophylla* Harms | A | BE |
| Fabaceae | *Daniellia oliveri* (Rolfe) Hutch. & Dalziel | W | BE |
| Fabaceae | *Desmodium adscendens* (Sw.) DC. | F | GA |
| Fabaceae | *Desmodium barbatum* (L.) Benth. | A | SA |
| Fabaceae | *Desmodium ramosissimum* G.Don | W | GH |
| Fabaceae | *Desmodium velutinum* (Willd.) DC. | F | BE |
| Fabaceae | *Distemonanthus benthamianus* Baill. | F | GA |
| Fabaceae | *Erythrina senegalensis* DC. | F | BE |
| Fabaceae | Fabaceae sp. | W | BE |
| Fabaceae | Fabaceae sp. | F | BE |
| Fabaceae | *Indigofera suffruticosa* Mill. | E | CR |
| Fabaceae | *Mimosa myriadena* (Benth.) Benth. | W | SA |
| Fabaceae | *Mimosa pudica* L. | W | SA |
| Fabaceae | *Mimosa quadrivalvis var. leptocarpa* (DC.) Barneby | F | BE |
| Fabaceae | *Mucuna* sp. | F | BE |
| Fabaceae | *Parkia biglobosa* (Jacq.) G.Don | W | BE |
| Fabaceae | *Pentaclethra macrophylla* Benth. | E | GA |
| Fabaceae | *Prosopis africana* (Guill. & Perr.) Taub. | W | BE |
| Fabaceae | *Pseudopiptadenia suaveolens* (Miq.) J.W. Grimes | W | SA |
| Fabaceae | *Pterocarpus erinaceus* Poir. | W | BE |
| Fabaceae | *Pterocarpus santalinoides* DC. | A | BE |
| Fabaceae | *Pterocarpus soyauxii* Taub. | W, F | GA |
| Fabaceae | *Senna alata* (L.) Roxb. | A | CR, GA |
| Fabaceae | *Senna chrysocarpa* (Desv.) H.S.Irwin & Barneby | A | AU |
| Fabaceae | *Senna occidentalis* (L.) Link | W  E  A  F | BE  SA, AU  SA  BE |
| Fabaceae | *Senna quinquangulata* (Rich.) H.S.Irwin & Barneby | W | SA |
| Fabaceae | *Stylosanthes fruticosa* (Retz.) Alston | W | GH |
| Huaceae | *Afrostyrax lepidophyllus* Mildbr. | W | GH |
| Hypericaceae | *Harungana madagascariensis* Lam. ex Poir. | A | GA |
| Hypericaceae | *Vismia guianensis* (Aubl.) Pers. | W | SA |
| Hypericaceae | *Vismia macrophylla* Kunth | W | SA, AU |
| Lamiaceae | *Clerodendrum capitatum* (Willd.) Schumach. & Thonn. | A | BE |
| Lamiaceae | *Clerodendrum* sp. | F | GA |
| Lamiaceae | *Hyptis atrorubens* Poit. | A | AU |
| Lamiaceae | *Hyptis suaveolens* (L.) Poit. | A | BE |
| Lamiaceae | *Ocimum americanum* L. | W  E  A, F | GA  GH  BE |
| Lamiaceae | *Ocimum campechianum* Mill. | E  A | SA  AU |
| Lamiaceae | *Ocimum gratissimum* L. | W  A | BE  BE, GA |
| Lamiaceae | *Premna quadrifolia* Schumach. & Thonn. | A | BE |
| Lauraceae | *Cassytha filiformis* L. | F | BE |
| Loganiaceae | *Spigelia anthelmia* L. | A | SA |
| Loranthaceae | *Oryctanthus alveolatus* (Kunth) Kuijt | W | SA |
| Loranthaceae | *Oryctanthus florulentus* (Rich.) Tiegh. | W  W, A | SA  AU |
| Loranthaceae | *Phthirusa pyrifolia* (Kunth) Eichler | W | SA |
| Loranthaceae | *Phthirusa stelis* (L.) Kuijt | W | SA |
| Loranthaceae | *Struthanthus syringifolius* (Mart.) Mart. | W | AU |
| Lycopodiaceae | *Lycopodiella cernua* (L.) Pic. Serm. | E  W | SA  BE |
| Lygodiaceae | *Lygodium volubile* Sw. | W | AU |
| Malpighiaceae | *Hiraea faginea* (Sw.) Nied. | W | AU |
| Malpighiaceae | *Stigmaphyllon sinuatum* (DC.) A.Juss. | W | SA |
| Malvaceae | *Abelmoschus esculentus* (L.) Moench | F | BE |
| Malvaceae | *Cola caricifolia* (G.Don) K.Schum. | W | GH |
| Malvaceae | *Cola nitida* (Vent.) Schott & Endl. | F | GH |
| Malvaceae | *Cola* sp. | F | GA |
| Malvaceae | *Gossypium barbadense* L. | W, A  E, A | AU  SA |
| Malvaceae | *Grewia* sp. | W | GA |
| Malvaceae | *Hibiscus acetosella* Welw. ex Hiern | A | GA |
| Malvaceae | *Hibiscus* sp. | F | GA |
| Malvaceae | *Hibiscus* sp. CF | W | GA |
| Malvaceae | *Cola gigantea* A. Chev. | A | BE |
| Malvaceae | *Duboscia macrocarpa* Bocq. CF | W | GA |
| Malvaceae | *Sida acuta* Burm.f. | W | GA |
| Malvaceae | *Sida linifolia* Juss. ex Cav. | E | GH |
| Malvaceae | *Waltheria indica* L. | W, E, A  W | SA  AU |
| Marantaceae | *Ischnosiphon arouma* (Aubl.) Körn. | W | AU |
| Marantaceae | *Ischnosiphon gracilis* (Rudge) Körn. | W, A | AU |
| Marantaceae | *Ischnosiphon puberulus* Loes. | W, A | AU |
| Melastomataceae | *Nepsera aquatica* (Aubl.) Naudin | W, A  A | AU  SA |
| Meliaceae | *Carapa guianensis* Aubl. | A, F  A | AU  CR |
| Meliaceae | *Khaya senegalensis* (Desv.) A.Juss. | W, A | BE |
| Meliaceae | *Turraea heterophylla* Sm. | E | GH |
| Menispermaceae | *Abuta grandifolia* (Mart.) Sandwith | W | AU |
| Moraceae | *Ficus glumosa* (Delile) | W | BE |
| Moraceae | *Ficus schumacheri* (Liebm.) Griseb. | E | SA |
| Moraceae | *Ficus* sp. | E | GH |
| Moraceae | *Milicia excelsa* (Welw.) C.C.Berg | W, F | BE |
| Musaceae | *Musa* spp. | W  W, A  A, F | SA  AU  GA |
| Myristicaceae | *Pycnanthus angolensis* (Welw.) Warb. | A, F | GA |
| Myristicaceae | *Virola surinamensis* (Rol. ex Rottb.) Warb. | W | SA |
| Myrtaceae | *Campomanesia aromatica* (Aubl.) Griseb. | W  E | AU  SA |
| Myrtaceae | *Campomanesia grandiflora* (Aubl.) Sagot | E | SA |
| Myrtaceae | *Eugenia patrisii* Vahl | W | SA |
| Myrtaceae | *Eugenia* sp. | W, A | SA |
| Myrtaceae | *Myrciaria floribunda* (H. West ex Willd.) O. Berg | W, A | SA |
| Myrtaceae | *Psidium guajava* L. | E, A | AU |
| Myrtaceae | *Syzygium guineense* (Willd.) DC. | A | BE |
| Nyctaginaceae | *Boerhavia diffusa* L. | W | GH |
| Ochnaceae | *Lophira lanceolata* Tiegh. ex Keay | W | BE |
| Oleaceae | *Schrebera arborea* A.Chev. | F | BE |
| Onagraceae | *Ludwigia decurrens* Walter | A | AU |
| Orchidaceae | *Vanilla heterolopha* Summerh. | W | GA |
| Papaveraceae | *Argemone mexicana* L. | F | BE |
| Passifloraceae | *Barteria fistulosa* Mast. | W, A, F | GA |
| Pedaliaceae | *Ceratotheca sesamoides* Endl. | F | BE |
| Pedaliaceae | *Sesamum indicum* L. | E | CR |
| Peraceae | *Chaetocarpus africanus* Pax | F | GA |
| Phyllanthaceae | *Bridelia ferruginea* Benth. | W, A | BE |
| Phyllanthaceae | *Hieronyma alchorneoides* Allemão | A | AU |
| Piperaceae | *Peperomia pellucida* (L.) Kunth | E  A | SA, AU, CR  SA |
| Piperaceae | *Piper aduncum* L. | W, E, A | AU |
| Piperaceae | *Piper arboreum* Aubl. | W | SA |
| Piperaceae | *Piper avellanum* (Miq.) C. DC. | W | SA |
| Piperaceae | *Piper bartlingianum* (Miq.) C. DC. | W | SA |
| Piperaceae | *Piper guineense* Schumach. & Thonn. | W, F | BE |
| Piperaceae | *Piper hispidum* Sw. | W | SA |
| Piperaceae | *Piper marginatum* Jacq. | E  A | SA, AU  AU, CR |
| Plantaginaceae | *Scoparia dulcis* L. | W  E  A | AU, BE, GA  SA, CR  SA, CR |
| Poaceae | *Bambusa vulgaris* Schrad. | A | GA |
| Poaceae | *Cymbopogon citratus* (DC.) Stapf | A | SA |
| Poaceae | *Eleusine indica* (L.) Gaertn. | W  E | SA, AU  SA, AU, CR |
| Poaceae | *Imperata brasiliensis* Trin. | W | AU |
| Poaceae | *Olyra latifolia* L. | W | SA |
| Poaceae | *Oryza sativa* L. | W  E | SA, AU  CR |
| Poaceae | *Paspalum conjugatum* P.J. Bergius | W | SA, AU |
| Poaceae | *Saccharum officinarum* L. | E  A | CR  SA, CR |
| Poaceae | *Zea mays* L. | E  F | CR  AU |
| Polygalaceae | *Carpolobia alba* G.Don | F | GA |
| Polygonaceae | *Coccoloba* sp. | W | SA |
| Polyporaceae | *Pycnoporus sanguineus* (L.) Murill | A | GA |
| Portulacaceae | *Portulaca oleracea* L. | W  A | AU  SA, AU |
| Pteridaceae | *Acrostichum aureum* L. | W | BE |
| Pteridaceae | *Pityrogramma calomelanos* (L.) Link | A | SA |
| Rapateaceae | *Saxofridericia acuelata* Körn. | W | AU |
| Rhamnaceae | *Maesopsis eminii* Engl. | A | GA |
| Rubiaceae | *Chassalia kolly* (Schumach.) Hepper | F | BE |
| Rubiaceae | *Duroia aquatica* (Aubl.) Bremek. | E | SA |
| Rubiaceae | *Gardenia ternifolia* Schumach. & Thonn. | W | BE |
| Rubiaceae | *Geophila repens* (L.) I.M.Johnst. | W | GH |
| Rubiaceae | *Oldenlandia affinis* (Roem. & Schult.) DC. | F | BE |
| Rubiaceae | *Oldenlandia herbacea* (L.) Roxb. | A | AU |
| Rubiaceae | *Psychotria capitata* Ruiz & Pav. | W | SA |
| Rubiaceae | *Psychotria* sp. | W | SA |
| Rubiaceae | *Psydrax palma* (K.Schum.) Bridson | F | GA |
| Rubiaceae | *Rubiaceae* sp. | W | GA |
| Rubiaceae | *Sabicea calycina* Benth. | W | GH |
| Rubiaceae | *Sarcocephalus latifolius* (Sm.) E.A.Bruce | A | BE |
| Rubiaceae | *Spermacoce verticillata* L. | A | AU |
| Rubiaceae | *Uncaria guianensis* (Aubl.) J.F.Gmel. | A | AU, CR |
| Rutaceae | *Citrus aurantiifolia* (Christm.) Swingle | A | GA |
| Rutaceae | *Citrus limon* (L.) Osbeck | F | AU |
| Rutaceae | *Clausena anisata* (Willd.) Hook.f. ex Benth. | A | BE |
| Sapindaceae | *Allophylus africanus* P.Beauv. | A | GH |
| Sapindaceae | *Paullinia pinnata* L. | W  E  A | SA, AU, CR, GH  AU, GH  SA, AU, CR |
| Sapindaceae | *Vouarana guianensis* Aubl. | W | SA, AU |
| Sapotaceae | *Baillonella toxisperma* Pierre | A | GA |
| Sapotaceae | *Vitellaria paradoxa* C.F.Gaertn. | W | BE |
| Siparunaceae | *Siparuna guianensis* Aubl. | A | SA, AU |
| Smilacaceae | *Smilax schomburgkiana* Kunth | W | AU |
| Solanaceae | *Capsicum annuum* L. | E  A, F | SA  GA |
| Solanaceae | *Nicotiana tabacum* L. | A  F | GA  BE |
| Solanaceae | *Physalis angulata* L. | A | BE |
| Solanaceae | *Schwenckia americana* L. | A, F | BE |
| Solanaceae | *Solanum americanum* Mill. | A | SA |
| Urticaceae | *Cecropia obtusa* Trécul | W, A | SA |
| Urticaceae | *Cecropia peltata* L. | W | SA, GA |
| Urticaceae | *Cecropia sciadophylla* Mart. | W  E | SA  CR |
| Urticaceae | *Laportea aestuans* (L.) Chew | W | SA |
| Urticaceae | *Myrianthus arboreus* P.Beauv. | W, F | GA |
| Verbenaceae | *Lantana camara* L. | W  E  A | GH  SA, AU  BE |
| Verbenaceae | *Lippia alba* (Mill.) N.E.Br. ex Britton & P.Wilson | E | SA, AU |
| Verbenaceae | *Lippia multiflora* Moldenke | A | BE |
| Verbenaceae | *Stachytarpheta cayennensis* (Rich.) Vahl | W, A  E | SA, AU  AU |
| Verbenaceae | *Stachytarpheta jamaicensis* (L.) Vahl | W, A | SA |
| Viscaceae | Viscaceae sp. | W | SA |
| Vitaceae | *Cissus aralioides* (Welw. ex Baker) Planch. | W  F | GH  GA |
| Vitaceae | *Cissus dewevrei* De Wild. & T.Durand | A | GA |
| Vitaceae | *Cissus quadrangularis* L. | W | GH |
| Vitaceae | *Cissus* sp. | W | GH |
| Zingiberaceae | *Aframomum melegueta* K.Schum. | E  F | CR  AU, GH |
| Zingiberaceae | *Aframomum* spp. | A, F | GA |
| Zingiberaceae | *Renealmia alpinia* (Rottb.) Maas | E | AU |
| Zingiberaceae | *Renealmia floribunda* K.Schum. | E | AU |
| Zingiberaceae | *Zingiber officinale* Roscoe | A  F | GA  BE |
|  |  |  |  |
|  | indet.gueleguele (Fon) | W | BE |
|  | indet.gueleguele (Goun) | W | BE |
|  | indet.beho | F | BE |
|  | indet.adjedikoribo | A | BE |
|  | indet.atinman | F | BE |
|  | indet.avovoun | W | BE |
|  | indet.nagonintindo | A | BE |
|  | indet.owedo | A | BE |
|  | indet.DQ231 | W | BE |
|  | indet.ndibnjiho | A | GA |
|  | indet.ovindamey | A | GA |
|  | indet.abone | F | GA |
|  | indet.boboneyesam | F | GA |
|  | indet.menyak-abam | F | GA |
|  | indet.minbubu noir | A | GA |
|  | indet.ndengua | W | GA |
|  | indet.oswon | W | GA |
|  | indet.otzishelle | W | GA |
|  | indet.zin-unkun | W | GA |
|  | indet.TVA5948 | W | GH |
|  | indet.ahunyankua | E | GH |

Supporting Information Table S2 Matrix with plant species used per CBS and population, with indications of wild, weedy and domestic status, as well as occurrence per species.
